# Research involvement and engagement of adolescent and young adults in a cancer trajectory: a 5-year experience from a patient support facility at a university hospital

**DOI:** 10.1186/s40900-023-00464-z

**Published:** 2023-07-21

**Authors:** Helle Pappot, Sara Kaa Meier, Maiken Hjerming, Karin Piil, Signe Hanghøj

**Affiliations:** 1 Department of Oncology, Rigshospitalet, University Hospital of Copenhagen, Blegdamsvej 9, 2100 Copenhagen, Denmark; 2 User of AYA Support Facility ‘Kræftværket’, Rigshospitalet, University Hospital of Copenhagen, Copenhagen, Denmark; 3 Department of Haematology, Rigshospitalet, University Hospital of Copenhagen, Copenhagen, Denmark

## Abstract

**Background:**

The purpose of this case study is to describe how a vulnerable group of patients can be included in research. The activities, challenges, lessons learned, and reflections on the importance of patient involvement in research for 5 years (2016–2021) at the adolescent and young adult (AYA) cancer support facility, Kræftværket, are reported.

**Main body:**

A patient panel at Kræftværket, the Youth Panel has multiple aims, one of which is the ability to perform patient involvement in research, with the goal of achieving research of high quality. We here describe how Patient and Public involvement (PPI) can be customized to AYAs in a cancer trajectory, who face many challenges, including those in the physical, psychological, and social domains. During 2016–2021, Youth Panel meetings were planned every third month but interrupted during the COVID-19 pandemic. With a flexible structure and a dynamic panel including 10–15 varying AYAs in a cancer trajectory, engagement and involvement have been maintained. Eight research topics were investigated, seven of which were discussed and confirmed to be important by the Youth Panel. Out of eight topics, three were raised by patients, and five by researchers. One was not discussed due to COVID-19. Some of the challenges we have experienced were related to the flexible meeting structure and the differing expectations and priorities as well as the impact of COVID-19. However, we experienced that patient involvement is possible in the field of AYA oncology if a trusting environment is created. A key finding in our case study was, that without a national Danish PPI program and no defined international standard for PPI in AYA cancer research yet, we were able to give patients the possibility to give input to researchers on topics where research is missing.

**Conclusion:**

Here, we demonstrate how patient involvement in research has been performed at an AYA cancer facility, Kræftværket, during a 5-year period. We encourage others to perform patient-involving research, even in challenging populations. Ideally this must follow international standards for PPI in AYA cancer research when such exist to improve research with crucial insight from patients.

## Plain English resume

In this paper, we describe patient involvement in research at Kræftværket, a youth support center and social organization for AYAs in a cancer trajectory. The center is located at The University Hospital Rigshospitalet in Copenhagen, Denmark. Youth panel meetings are Kræftværkets’ most central patient involvement activity, and one of its aims is to facilitate high-quality patient-initiated research. AYA cancer patients are a vulnerable group facing huge psycho-social challenges and symptoms that make normal functioning difficult. Therefore, the youth panel is designed to be flexible in its structure, so participants do not have to commit themselves as permanent members. The youth panel meets four times a year, and during the period 2016–2021, it has been involved in eight research topics. Challenges include the flexible meeting structure, different expectations, and priorities as well as the impact of COVID-19. However, patient involvement has been possible because of a trusting environment with strong nurse-led support for the participants. We hope to encourage others to argue for and carry out meaningful patient-involving research to improve tomorrow’s quality of AYA cancer care.

## Background

In Denmark, approximately 600 AYAs in the age group 15–29 years are diagnosed with cancer each year. In descending order, the most common cancers for AYAs between the ages of 15 and 29 in Denmark are malignant melanoma, testicular cancer, cervical cancer, thyroid cancer, lymphoma, leukaemia, central nervous system cancer, and breast cancer [1, 2]. Rigshospitalet is a tertiary general university hospital, which, in contrast to the service for children in the paediatric department, has no dedicated adolescent-specific wards [3]. Annually, in the Copenhagen region, approximately 200 AYAs receive new cancer diagnoses, with the majority receiving treatment at Rigshospitalet, University Hospital of Copenhagen, alongside patients referred from other parts of Denmark [2]. To accommodate an unmet need both within physical, psychological, and social domains of the AYA patient population, a supportive initiative was created in 2015 for AYAs aged 15–29 years in a cancer trajectory at Rigshospitalet [3].

The supportive initiative for adolescents and young adults in a cancer trajectory is named Kræftværket. The primary objective of Kræftværket is to provide youth-friendly support, including adolescent-friendly facilities, resources, and activities. As Kræftværket provides services to both paediatric (patients aged 15–18 years) and adult departments (patients aged 19–29 years), a network has been created between patients who would otherwise not interact. The initiative is managed by a Youth Coordinator (clinical nurse-specialist dedicated to Kræftværket and its activities) and five specifically trained nurses: Youth Ambassadors [3], who have undergone basic education in PPI and remained the same throughout the study period.

Kræftværket allows AYAs in a cancer-trajectory (on treatment and in follow-up, including survivors) to connect with peers and enables the active participation and engagement of AYAs throughout the initiative. Both in-Kræftværket and out-of-hospital activities, e.g., dinners, music events, etc., for patients together with the youth coordinator and youth ambassadors, creates new relations and an including acknowledging and safe environment. Other objectives of Kræftværket are to develop multidisciplinary collaboration across adolescent medicine, paediatric, and adult cancer departments; to incorporate knowledge sharing, teaching, and involvement of relevant health professionals (e.g., nurses, doctors, physiotherapists); to coordinate political and administrative activities surrounding AYAs in a cancer trajectory; and to continuously improve quality of care and further research in the field of AYA cancer care [3].

In recent years, Patient and Public Involvement (PPI) has been recommended by funding agencies, especially in the United States of America, Canada, and the United Kingdom [4–6]. The purpose of involvement and engagement is to perform research of better quality, but it is also seen as a human right aiming at giving the public a say in publicly funded research [7]. Yet no international standard for patient involvement exists within the AYA cancer group, but recently a European Collaborative Workshop reported disparity in patient and parent involvement and engagement (PPIE) activities in childhood, adolescent and young adult cancer research across Europe and a need for European-level definition of PPIE for paediatric oncology [8]. However, unique experiences over time on involving young people in research exist from e.g., the British BRIGHTLIGHT study, evaluating teenage and young adult cancer services in England [9, 10]. A reason for sparse experience and evidence within the AYA cancer area can be, that not all patient groups might be equally likely to engage and be involved, but even within diagnosis influencing cognitive functions, this has been proven feasible [11]. For AYAs in a cancer trajectory, some special issues are to be considered when aiming at involving patients in research; AYAs face many challenges and have unmet needs, including those under physical, psychological, and social domains, and serious disease interrupts a critical period of physical and personal development, where relationships, academic, and professional careers, and planning for the future have a significant level of importance [12–14]. More informal structures for research involvement, such as a dynamic youth panel, can be used to accommodate the needs of this patient group if they should be involved in research. When the present initiative was initiated, Denmark had no structure for PPI and no international standard for PPI in AYA cancer research was available [8]. In parallel with the development of PPI initiatives in Denmark [15] and international activities in the field, we investigated how AYAs in a cancer trajectory can have influence on cancer research in our setup.

The aim of this case study is to describe how a vulnerable group of patients can be successfully included in research in real-life. The activities, challenges, lessons learned, and reflections on the importance of patient involvement in research for 5 years (2016–2021) at an AYA cancer support facility are reported.

## Activities

When Kræftværket was created as a patient-involving cocreation project in 2015, research was initially not defined as a focus point. Fastly, it became evident that the users of Kræftværket raised important issues, of which some could not be spontaneously solved because of a lack of knowledge in certain problem areas. A steering committee of Kræftværket, comprising representatives from different departments treating AYAs with cancer, leaders and the youth coordinator decided to attach a research group to Kræftværket. The research group comprises researchers on haematology, oncology, paediatric oncology, youth and nursing research. This group of researchers should act on input from the patients and aim at patient-involving research. Since 2016, the research group has worked according to this action plan, and patient involvement has been a cornerstone in the performed research activities. Aiming at continuous patient involvement not only in research but in all aspects of the AYA cancer trajectory, it had from the beginning also been decided to have a patient panel at Kræftværket, the youth panel. Here, the activities, challenges, lessons learned, and reflections on the importance of patient involvement in research at Kræftværket are described from the perspective of a patient, three researchers and a youth coordinator.

### Recruitment

Denmark has no tradition for systematic patient involvement or agreed framework here for, and at the time, where our initiative was ongoing, the hospital did not have a patient council or other patient-involving structures. Many other researchers have demonstrated and discussed how patients and other users can be recruited per application and selected to form a user panel with broad representation across age groups, gender, diagnosis, etc. [16]. In this case, we choose a different approach, seeing the fact that AYAs in a cancer trajectory are a certain vulnerable group going through a transition phase of their life [12], where priorities might be differing over time and adherence to a patient panel can be difficult. Given the life circumstances for AYAs in a cancer trajectory, we choose an approach with a flexible patient panel, the youth panel, comprising no permanent members but open to everyone within the target group. The AYAs approached were characterized by focusing more on youth problems common for the group than on specific problems related to diagnosis and in general showed a high degree of altruism.

### Youth panel

A youth panel typically included between 10 and 15 AYA participants representing both patients with haematological and oncological diseases and a variety of malignant diagnoses. Typically, some of the patients attending would be in active antineoplastic therapy, but patients in follow-up (survivors) or palliative care could also attend.

Youth panel meetings were scheduled four times a year. Apart from AYAs in a cancer trajectory, and doctors, Youth Ambassadors, and the Youth Coordinator were also invited. Youth panel meetings were announced on the Kræftværkets closed Facebook group. In the announcement, the agenda for the youth panel meeting was shown. All users of Kræftværket were welcome to sign up for the youth panel meeting, but this should be done prior to the activity. Normally 1–3 issues on the agenda were related to research.

### Meetings

Meetings were planned at the Kræftværkets facility in the hospital, and snacks and a meal were served to all participants at every meeting. A welcoming and warm atmosphere was created by the youth coordinator, with whom all participants were familiar. A user said about the meetings: ‘I felt seen, heard and understood’ (female, 29 years, youth panel participant).

At the meetings, a voluntary patient representative was given the role of moderator. All meetings started with an introduction round and welcoming, and the aim of the actual youth panel meeting was highlighted by the youth coordinator. At least one researcher was present at each meeting, but one or more researchers could be invited to present ideas or discuss subjects related to AYAs in a cancer trajectory, the researchers were in general not treating physicians, only one researcher (HP) had treatment responsibility for in average less than 5 percent of the patients in the Youth panel. The youth coordinator was responsible for the minutes from the meeting. Subjects in the youth panel meetings were not limited to research issues, and the meetings had a relaxed atmosphere. All kinds of subjects could be brought up, e.g., practical issues, peer-to-peer advice, and ideas for new activities. Often, challenges were discussed peer-to-peer with the opportunity to obtain input from health care professionals. An example could be a discussion between young women on experiences with fertility counselling leading to a question to health care professionals and researchers about guidelines for fertility counselling. In this case, the discussion ended with a wish from the patients to investigate fertility counselling in young, female cancer patients to determine how to improve this service. The topic raised by patients was then transferred into a research question and acted on by the researchers, and primary results from the research project have been published [17].

During the 5-year period, the COVID-19 pandemic made periodic physical meetings impossible. In total 14 meetings were taking place in the 5-year period. In that period, virtual calls were planned and offered for psychological support biweekly; however, these meetings were more formal and had fewer participants and the meetings had very little focus on research but made it possible to discuss urgent research issues.

### Website and communication

To ensure easily accessible information about Kræftværket, an explanation about the youth panel and an introduction to the youth coordinator and the youth ambassadors a webpage was created [18]. This website was designed according to the hospitals’ communication guidelines. On the website links to Kræftværkets, an open Facebook page and Instagram account could be found, which could be used to communicate research activities and new findings not only to the patients but also to the public. The website also gave the possibility to show examples via links on patient involvement in research, which was, among others, used when an app for AYAs in a cancer trajectory was cocreated [19].

Minutes from the youth panel meetings were always available in the closed Facebook group of Kræftværket.

### Engagement with Kræftværket’s research and activities

**Fig. 1:**
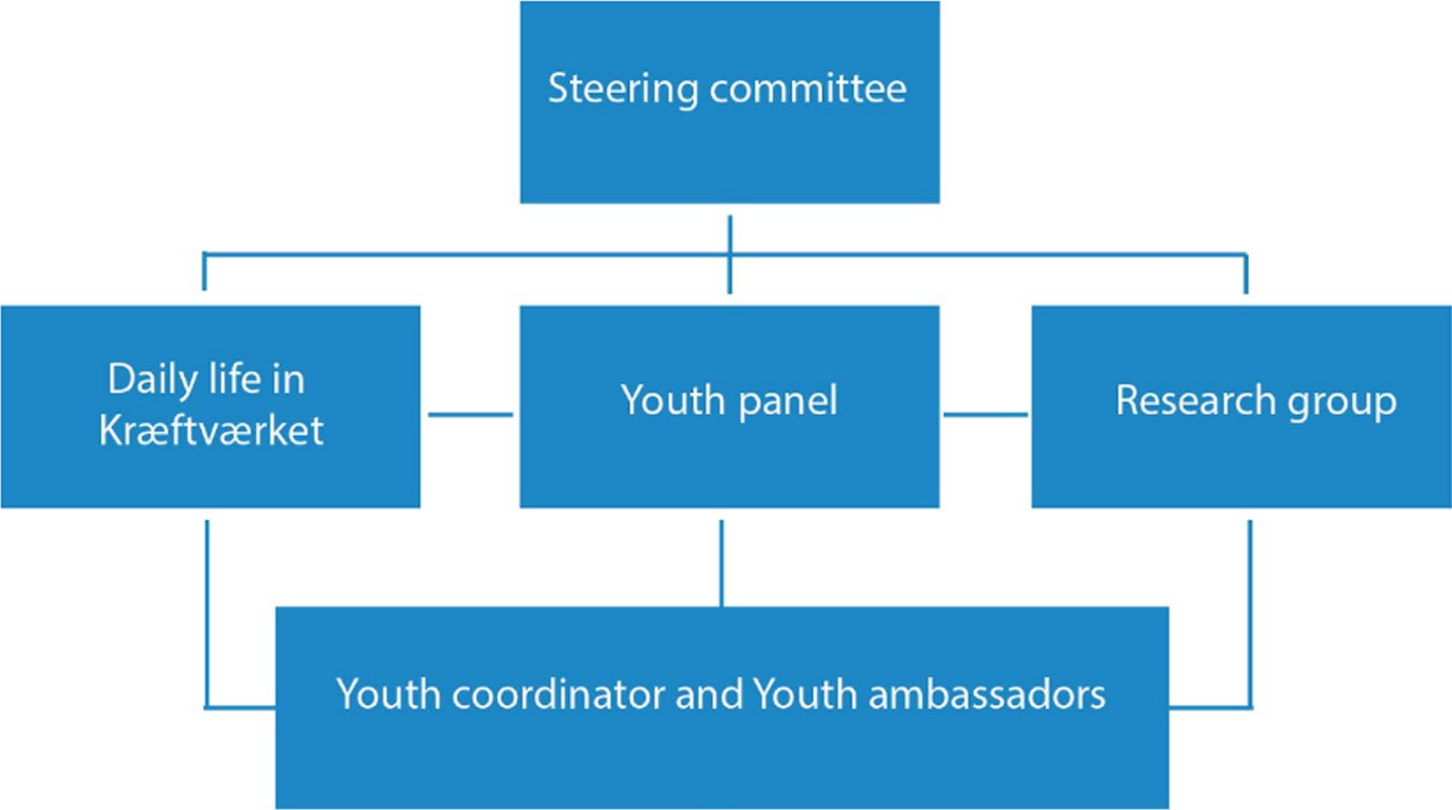
Organization of Kræftværket showing communication flow in between different participants

Since both the youth panel and the research group were formally attached to Kræftværket (see Fig. 1), dissemination of the possibility for patient involvement in research projects seemed easy. The youth coordinator always attended both youth panel meetings and research group meetings together with at least one medical doctor/researcher. A fixed item on the agenda at the research meetings was a resume of the most recent youth panel meeting. In this way, ideas, suggestions and possible research topics were transferred from patients to researchers. All ideas brought up by researchers should, if possible, be discussed with the youth panel before the initiation of any project and if the project was a consequence of an initial patient idea. This way of working and thinking trained the researchers in being focused on patient involvement early in the research project and preferably at the idea phase. Table 1 shows patient-involving research projects at the supportive facility for adolescents and young adults in a cancer trajectory, Kræftværket, during a 5-year period, 2016–2021. Three (App development, Cognitive challenges and Fertility) out of 8 research topics were raised by the patients and later transformed into research projects. A project during the COVID-19 pandemic on the effect of isolation on AYAs in a cancer trajectory was not discussed with the panel due to the close-down of all Kræftværket activities when the project was run [20]. The projects concerning patient involvement was not discussed individually but were for some indirectly accepted by the youth panel because they were part of other patient-involving activities, which were previously discussed at the youth panel, e.g., the app development project also had a project investigating the experience of being involved in app development and other patient activities as a user [21].

**Table 1:** Patient-involving research projects at a supportive facility for adolescents and young adults with cancer during a 5-year period, 2016–2021

Topics	Topic raised by	Topic discussed and problem confirmed by patient-panel	Type of study	Publications
App development	Users	x	Cocreation	[24, 28]
Patient-involvement	Researchers	(x)	Qualitative studies	[21, 25, 26, 28]
Quality of life	Researchers	x	Observational study	[36]
Covid19	Researchers	–	Qualitative study	[20]
Living alone	Researchers	x	Qualitative study	Study in progress
Cognitive challenges	Users	x	Observational study	[37]
Fertility	Users	x	Register study Qualitative study	[17]
Monitoring	Researchers	x	Observational study	[34]

– = not discussed due to Covid19, (x) = discussed as secondary endpoints of other projects e.g., the app development

### Evaluation

From the start of the research groups’ work with patient involvement, there was awareness of the need to evaluate the process. We found that a more in-depth evaluation was necessary. A combination of interviews and questionnaires was not chosen since the existing tool, the Patient Engagement in Research Scale (PEIRS-22) [22], was not available in Danish at that time. Furthermore, we previously experienced a ceiling effect when developing evaluation questionnaires in cocreation with patients in a breast cancer setting [23]. Therefore, different aspects of the involvement process were evaluated via qualitative research projects, which were performed as either adding on to specific projects [24] or describing experiences of involvement processes during participation in cancer service user initiatives [21] as well as the impact of service patient involvement from the perspective of adolescents and young adults in a cancer trajectory [25]. Additionally, the reason for helping others by taking part in service user design was investigated [26]. This somewhat extensive approach was taken to learn more about patient-involving processes in the vulnerable group of AYAs in a cancer trajectory.

## Challenges

During the 5-year period with patient involvement in research projects at an AYA support facility, Kræftværket, more challenges were met, the most important of which were:

### COVID-19

In March 2020, when COVID-19 was declared a global pandemic, patient involvement activities became more difficult. Youth panel meetings did not initially take place, and a new workflow for interaction with patients without physical meetings had to be introduced. Planned activities such as youth panel meetings were rescheduled to virtual meetings with less or no focus on research, and patient-involving specific projects had to use telephone interviews instead of physical meetings, whereas other projects simply had to be postponed until physical meetings were safe and allowed by health authorities. This period was challenging to the AYAs, and we performed a qualitative project to highlight the special problems influencing their life in this period of isolation [20]. Connections between AYAs and the youth coordinator were partly maintained by offering walk-and-talks on a one-to-one basis when possible.

### Meeting structure

The technical issues of performing virtual meetings instead of in-person meetings in this younger patient group were limited, but fewer people wanted to participate when the meetings were virtual, sometimes as few as 2–4 persons.

From the beginning, when introducing in-person meetings we choose a flexible youth panel, with each meeting being open to new members of the panel and some only participating one or a few times. The disadvantage of this setup was that information often had to be repeated, since not all had taken part in prior meetings; at the same time, it helped others recall discussions they had already taken part in. The advantage of the flexible youth panel was that the panel and the researchers were continuously challenged by newcomers, and new insights and topics were brought up. At some point, researchers could become frustrated because an initial youth panel had confirmed a research topic initially, but at a later point, a transformed panel could be more critical of the project. Taken together, this fact led to the optimization of many research projects, e.g., more concise inclusion criteria for a specific project.

### Differing expectations and priorities

The youth panel is a collaboration between young people living with cancer or being survivors in a follow-up program, health care professionals with a special focus on AYAs in a cancer trajectory (youth coordinator and youth ambassadors) and doctors/researchers. These participants all strive to improve the health and quality of life of young people living with or after cancer. However, the process through which to achieve these goals could be a discussion point in the panel, while researchers often had longer-term academic goals such as grants and publications, patients preferred fast action achieved through, e.g., addressing the press for attention to specific problems. Through bilateral respect and communication in lay-person language, patients and researchers over time got a better understanding of different approaches to the same subject. Especially for researchers it was a learning process to simultaneous be confronted with the patients’ frustrations and extract valuable inputs for a research process.

Because adolescents and young adults are in a transition phase, many different activities have to be prioritized (friends, education, family, new romantic relations, problematic housing, etc.), it was difficult to maintain a continuous group of people in the youth panel, which sometimes made it difficult to fulfil the researchers’ expectations but was a prerequisite and seemed to be problem-free for the patients, who dropped in and out of the panel in the 5-year period.

## Lessons learned

During the 5-year period, we tried to gain growing knowledge on research engagement and involvement among AYAs in a cancer trajectory. In parallel with performing research projects, we have investigated the reasons for and impact of being involved in research/cocreation projects [25, 26]. From these investigations, we have learned that AYAs in a cancer trajectory are very altruistic and are willing to use their own experiences in peer-to-peer relations. Other themes that we have learned are as follows.

### Develop a trusting environment

The youth coordinator of Kræftværket played an essential role in the process. The AYAs in a cancer trajectory already knew and had trust in this key person, and mutual respect existed between the AYAs and the coordinator. The engagement of the youth coordinator in the youth panel and in the research has been essential for creating a trusting environment. However, every time a new researcher was presented in the youth panel, this new person had to be reminded about the importance of valuing different perspectives and giving equal opportunity for feedback, and it could take some time before a new researcher had developed reciprocal relationships including these values.

To create a trusting environment with good relationships, we found it valuable to have informal welcome sessions where everyone could catch up, socialize and give support to each other. When the meetings were ongoing, it was important to give many opportunities to the youth panel participants to share their experiences, insights and meanings. It was observed that participants who knew each other better had a higher likelihood of speaking up, but by creating an atmosphere of trust, which was facilitated both by the youth coordinator and more experienced members of the panel, the voice of all participants was heard.

### Provide support

When we started the research involving process, none of the AYAs in a cancer trajectory involved had experience with being involved in research, but some of the volunteers who joined the process were researchers themselves or had educations within health care, giving them different insights than other participants. As time evolved, the youth panel became a mixture of experienced patients who knew about research involvement and newcomers. In this group, the youth coordinator, and the experienced participants, both on a peer-to-peer level and in the group, supported new participants and shared their knowledge and experiences with involvement, leading to a continuous back-up from patients to the initiative.

In Denmark, there is no formalized education of patients who take part in research, as is known from, e.g., the United Kingdom [27]. All participants are individuals who participate with their unique background and insight. During the 5-year period, the user-involving field in Denmark has developed, and alongside our process, many hospitals have established patient councils, involving both patients and relatives, in which members participate based on application and evaluation and where diversity is secured. The co-author, SKM, is a patient-representative in Righospitalets Research-council.

### Flexibility

Participants in the youth panel had different challenges in participating. Geographic location and transportation could, for some, be an issue to manage scheduled meetings; for others, work or study obligations could stand in the way for participation, which is why meetings were always planned in the late afternoon followed by dinner to give as many as possible the opportunity to participate. It could have been hypothesized that virtual meetings would have had higher participation rates due to the more flexible setup allowing meeting participation from home; however, it was our experience that AYAs in a cancer trajectory suffered from ‘virtual meeting fatigue’ during the pandemic and longed for physical meetings, leading to less participation in virtual meetings. This dilemma has been described by us and others under the theme of ‘social isolation’ [20]. Even though researchers were often busy and engaged in many activities at the hospital, it was our impression that they also preferred the physical meetings because they experienced the open, trustful atmosphere in the room leading to improved communication and respectful insight sharing.

The flexibility in the composition of the youth panel might seem challenging with respect to diverse representation, but this was overcome by a very open structure where all interested patients were invited to participate, and we have in more exact projects shown how a diversity of participants choose to take part in patient-involving activities [28]. In this way, representation by gender, diagnosis, geography, and age was randomly widespread.

### Acknowledge tensions and imbalances

Patients and researchers participating in the youth panel shared a common goal: improving the lives of AYAs in a cancer trajectory. Researchers and patients could, however, have different views on how to achieve this goal. While researchers are often driven by slow funding processes based on grant applications, followed by long research processes leading to a conclusion that can then, if meaningful, be implemented, the patients in some matters demand fast action. Unfortunately, as for education in research involvement, Denmark has no support program for system-level advocacy policy as in some other countries [29]. In our case, the youth coordinator was continuously available with legal and personal support to patients, who publicly tried to draw political attention to the special challenges faced by AYAs in a cancer trajectory.

As the participants in the youth panel had different experiences and background imbalances could occur between participants, it was observed that despite these differences, participants were acting very respectfully towards each other and valuing the insight of all at an equal level. Participants showed great patience with each other when, e.g., difficult research projects had to be repeatedly explained to secure understanding among all. Although some AYAs were challenged by cognitive impairment, the atmosphere at Kræftværket facilitated the inclusion of all individual viewpoints. We believe that the Youth coordinator played an important role, as facilitator of a safe atmosphere and stable relationships.

## Reflections on the importance of engagement

During the 5-year period where research engagement and involvement were performed in the supportive facility Kræftværket, different reflections on the importance of engagement arose. Here, we share some of the important themes:

### Creating meaning

Participants in the youth panel felt that their participation gave them a chance to be heard, and they were also keen on that their experiences might help others. It seemed as if the participation was empowering the patients. The participants experienced that their engagement in research, including the formation of new relationships with others, helping others, and having a voice, made sense. The meaningfulness of helping others is described based on our setup [26] but has also been raised by others [30]. A user said, ‘I remember that I was asked to review the minutes from a youth panel meeting written by one of the researchers. I did that during chemotherapy wearing ice-gloves. At that moment I felt appreciated and valuable—it made me extremely happy’ (female, 29 years, youth panel participant).

### Contributing to research

Others have described what happens, when people with lived experience of being AYA and having cancer get the chance to share experiences grounded in personal experience, this offers researchers new insights into issues and improves their understanding of health needs [31, 32]. Further, it has been stated by Forsythe et al. [33] that aspects of research studies can be improved through engagement, which includes feasibility, acceptability, and relevance. In our real-life experience of PPI an example hereof was a research project including a wearable device for monitoring vital signs. Only by the input from patients in the phase of designing the research protocol was it possible to design a wearable study, which was feasible for AYAs according to wear time [34]. Our AYA population also described how patients both experience involvement processes [21] and how they see the impact of service user involvement from their perspective [25].

### Identifying barriers to engagement

When researchers and patients are brought together and work together, it can help researchers to identify barriers for AYAs in a cancer trajectory to join research projects. Many good discussions emerged on the accessibility of information and the encouragement from patients to use lay-person language or visuals for better understanding. It was also very valuable with the researchers’ understanding of the AYAs’ life condition, making them respect financial and time limitations as well as limited resources for participation [35].

## Moving forward

Due to the flexible setup of our patient-involving initiative, we have experienced both fast success and more complex research processes. This 5-year period has led to new insights into the lives of AYAs in a cancer trajectory, insights that have helped researchers to perform research within the field with more focus on problems that matter to the individuals here and now but also qualify more complex projects to ensure enrolment in important studies in the future. At present, this patient-involving initiative is at a crossroads, where it must be decided if more formalized involvement is needed, as described by researchers in primarily English-speaking countries [35]. In collaboration with the Danish Cancer Society and other large patient organizations, a plan for possible education of patients to be involved in research must be discussed to ensure that involvement of patients is performed respectfully and with the intention of using patients’ insight to improve research.

## Conclusion

During the 5-year period 2016–2021, several youth panel meetings in our AYA cancer support facility have taken place. At these meetings, research involvement has always been an important issue, and throughout the period, a variety of AYAs have taken part in ensuring diversity in the insights shared on different topics. The setup we used has given patients the possibility to give input to researchers on topics that need more attention and where research is missing. Reporting this real-life experience of PPI for AYAs in a cancer trajectory shows how research originating from Kræftværket and co-work between researchers and patients has, among others, led to the implementation of a national app for AYAs in a cancer trajectory aiming at improving quality of life [36], the design of a research project aiming at improving fertility counselling for AYAs with cancer and new insights into social isolation among AYAs in a cancer trajectory [20]. We encourage other researchers to be willing to perform patient-involving research to improve their research with the help of patients’ insight but suggest that this is done according to international standards for PPI in AYA cancer research when such are available.

## Data Availability

Not applicable.
